# Imageless robotic total knee arthroplasty determines similar coronal plane alignment of the knee (CPAK) parameters to long leg radiographs

**DOI:** 10.1186/s42836-024-00231-9

**Published:** 2024-03-03

**Authors:** Adam I. Edelstein, Alexander D. Orsi, Christopher Plaskos, Simon Coffey, Linda I. Suleiman

**Affiliations:** 1 Department of Orthopaedic Surgery, Northwestern University Feinberg School of Medicine, Chicago, IL 60611 USA; 2 Clinical Research, Corin, Raynham, MA 02767 USA; 3 Department of Orthopaedics, Nepean Hospital, Penrith, NSW 2750 Australia

**Keywords:** Coronal plane alignment of the knee, Robotic assisted surgery, Total knee arthroplasty

## Abstract

**Background:**

The coronal plane alignment of the knee (CPAK) classification was first developed using long leg radiographs (LLR) and has since been reported using image-based and imageless robotic total knee arthroplasty (TKA) systems. However, the correspondence between imageless robotics and LLR-derived CPAK parameters has yet to be investigated. This study therefore examined the differences in CPAK parameters determined with LLR and imageless robotic navigation using either generic or optimized cartilage wear assumptions.

**Methods:**

Medial proximal tibial angle (MPTA) and lateral distal femoral angle (LDFA) were determined from the intraoperative registration data of 61 imageless robotic TKAs using either a generic 2 mm literature-based wear assumption (Nav_lit_) or an optimized wear assumption (Nav_opt_) found using an error minimization algorithm. MPTA and LDFA were also measured from preoperative LLR by two observers and intraclass correlation coefficients (ICCs) were calculated. MPTA, LDFA, joint line obliquity (JLO), and arithmetic hip-knee-ankle angle (aHKA) were compared between the robotic and the average LLR measurements over the two observers.

**Results:**

ICCs between observers for LLR were over 0.95 for MPTA, LDFA, JLO, and aHKA, indicating excellent agreement. Mean CPAK differences were not significant between LLR and Nav_lit_ (all differences within 0.6°, *P* > 0.1) or Nav_opt_ (all within 0.1°, *P* > 0.83). Mean absolute errors (MAE) between LLR and Nav_lit_ were: LDFA = 1.4°, MPTA = 2.0°, JLO = 2.1°, and aHKA = 2.7°. Compared to LLR, the generic wear classified 88% and the optimized wear classified 94% of knees within one CPAK group. Bland–Altman comparisons reported good agreement for LLR vs. Nav_lit_ and Nav_opt_, with > 95% and > 91.8% of measurements within the limits of agreement across all CPAK parameters, respectively.

**Conclusions:**

Imageless robotic navigation data can be used to calculate CPAK parameters for arthritic knees undergoing TKA with good agreement to LLR. Generic wear assumptions determined MPTA and LDFA with MAE within 2° and optimizing wear assumptions showed negligible improvement.

**Supplementary Information:**

The online version contains supplementary material available at 10.1186/s42836-024-00231-9.

## Introduction

The persistence of suboptimal satisfaction outcomes among patients following total knee arthroplasty (TKA) has led to the development of innovative surgical techniques [1–3]. Personalized alignment strategies in total knee arthroplasty have emerged as alternatives to neutral mechanical alignment [4, 5]. Kinematic alignment and related approaches aim to recreate native knee morphology by removing the amount of bone and cartilage that will be exactly replaced by the implant, with the intent of restoring natural kinematics and joint laxities [6, 7]_._ These personalized approaches have been reported to achieve improved balance, kinematics, and outcomes compared to mechanical alignment [8, 9].

Key to personalized alignment is the restoration of the pre-arthritic joint surfaces. The arithmetic hip-knee-ankle angle (aHKA) [10], providing information on the constitutional alignment of the lower limb irrespective of cartilage loss, and the joint line obliquity (JLO) together comprise the coronal plane alignment of the knee (CPAK) classification [11]. CPAK was developed using measurements made on long leg radiographs (LLR) and defines nine native knee phenotypes that can be targeted in a personalized TKA, based on a 3 × 3 grid organized by aHKA (varus, neutral, valgus) and JLO (apex distal, neutral, and apex proximal) [11].

Several strategies exist for intraoperative determination of native alignment and JLO, including the use of calipers and assumptions about the thickness of lost cartilage, custom cutting blocks, and robotic approaches with or without preoperative imaging [12–15]. The ability of CT-based robotics to define constitutional alignment and CPAK has been established [16–18], yet it remains unknown if imageless intraoperative robotic data can accurately describe CPAK parameters [19–23]. Furthermore, optimal cartilage wear assumptions to be used with imageless navigation data remain undefined.

The purpose of this study was to determine if intraoperative imageless robotic data can define the CPAK classification for arthritic knees at time of TKA. The goal was to investigate the accuracy of navigated CPAK parameters in comparison to CPAK parameters generated from LLR. Another goal was to define the cartilage wear assumptions for use in imageless robotics that would best approximate CPAK parameters determined from LLR. It was hypothesized that imageless robotic CPAK parameters would be in statistical agreement with LLR. Establishing the reliability of imageless robotic CPAK parameters would support the use of this operative modality for restoring native joint lines.

## Methods

Sixty-two robotic TKA procedures performed between February 2021 and November 2022 were retrospectively reviewed following approval from an independent institutional review board (WCG IRB No. 120190312 and Bellberry Ltd. No. 2020–08-764). Operations were performed by an experienced surgeon (SC) with over 10 years of experience with surgical robotics and navigation in TKA prior to this study. Inclusion criteria involved patients with end-stage osteoarthritis (Kellgren-Lawrence grade ≥ 3) having preoperative LLR and undergoing robotic-assisted primary TKA with the imageless OMNIBotics system (Corin Group, Cirencester, UK). Severe valgus deformities with marked bone loss were excluded (*n* = 1). Patients had an average age of 70 ± 9 years, an average BMI of 32 ± 7 kg/m^2^, were 63% female, had a preoperative coronal deformity of 3° ± 6° varus (range: 16° valgus to 17° varus), and a preoperative flexion contracture of 4° ± 7° (range: 16° hyperextension to 23° flexion).

### Radiographic measurements

**Fig. 1:**
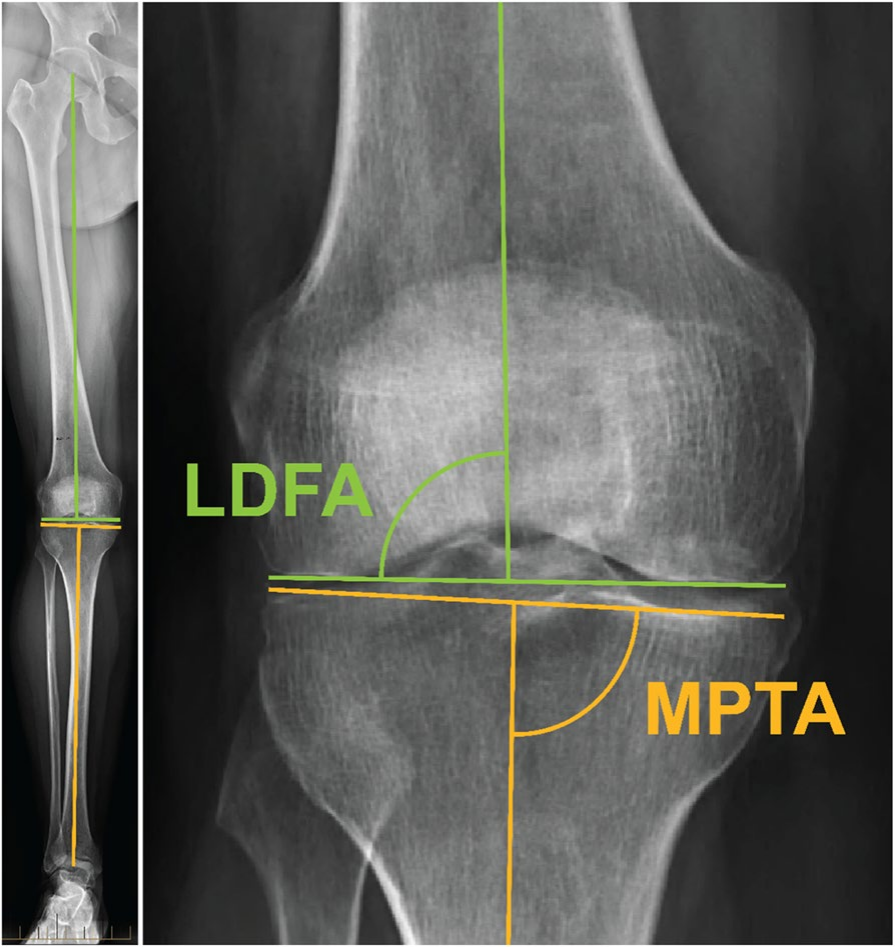
MPTA and LDFA measurement on long leg radiograph (LLR)

The mechanical axis (MA) of the tibia was defined as the line between the ankle center and the center of the tibial spines. The MA of the femur was defined as the line between the femoral head center and center of the intercondylar notch. The MPTA was defined as the angle between the tibial MA and the line between the most distal articulation points on the medial and lateral proximal tibial plateaus. The LDFA was defined as the angle between the femoral MA and the line between the most distal articulation points on the medial and lateral distal femoral condyles. Measurements were taken from preoperative radiographs post-hoc by an orthopaedic surgeon (AE), and a senior technology research engineer (AO) as shown in Fig. 1 [11].

### Robotic navigation measurements and wear assumptions

**Fig. 2:**
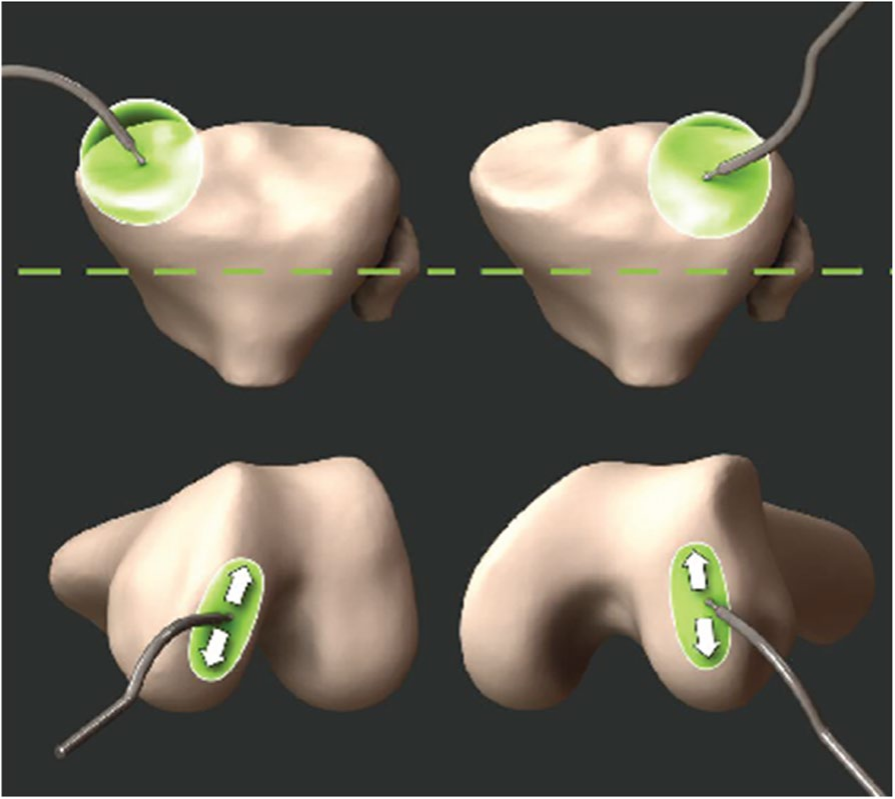
Intraoperative collection of proximal tibial (top) and distal femoral (bottom) landmarks. The proximal tibial points were selected by the surgeon while the most distal points on the femur were computed automatically using the femoral morphometric model

Landmarks were acquired using the robotic navigation system to mark the distal femoral condylar and proximal tibial condylar points, as seen in Fig. 2. For the medial tibial condylar landmark in the cases of medial cartilage wear, a medial tibial cartilage-to-bone tidemark point was used, which was in the same anterior-posterior (AP) plane as the lateral tibial condylar point, similar to Murgier and Clatworthy [24]. The lateral tibial condylar landmark remained in the mid-coronal plane even in cases of valgus knees with isolated posterolateral cartilage loss. Due to the imageless nature of the robotics system, a morphometric model of the distal femur is created by digitizing, or “painting”, points using the navigation probe [25]. The medial and lateral distal femoral condylar points were automatically calculated from this bone morph as the most distal points on each condyle along the direction of the femoral mechanical axis. These tibial and femoral landmarks were then used to calculate MPTA and LDFA by applying wear assumptions based on preoperative deformity, which was captured using the navigation system after registration. LDFA is measured relative to the mechanical axis of the femur, which was calculated as the line joining the kinematic center of the hip joint, as determined by circumduction of the hip joint [26], and the center of the distal femur, as landmarked by the surgeon. MPTA is measured relative to the mechanical axis of the tibia, which was calculated as the axis line joining the ankle center, as determined by the midpoint between the most extreme points on the medial and lateral malleoli, and the center of the proximal tibia, as landmarked by the surgeon [27, 28].

**Fig. 3:**
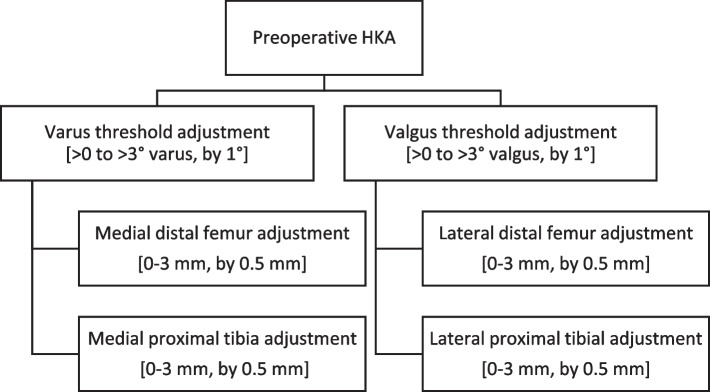
Range of deformity threshold and compartmental wear adjustments used to determine optimal wear correction (Nav_opt_)

Two wear assumptions were evaluated on the intraoperative data of robotic navigation system. First, a literature-based nominal wear correction (Nav_lit_) of 2 mm on the medial and lateral distal femur and on the proximal tibia was assessed for preoperative varus (medial wear) and valgus (lateral wear) deformities ≥ 3° HKA, as described by other authors [6, 24, 29]. Secondly, an optimized wear assumption (Nav_opt_) was determined by iterating through the range of parameters described in Fig. 3, and selecting the combination which minimized the root mean square error (RMSE) relative to LLR_mean_ for MPTA and LDFA. The Nav_opt_ parameters are shown in Table 1.

**Table 1:** Wear assumptions for both the Nav_lit_ and Nav_opt_ models

**Preoperative deformity**	**Wear assumption**	Nav_lit_	Nav_opt_
Varus	Varus HKA threshold (°)	3	1
	Medial distal femoral adjustment (mm)	2.0	2.0
	Medial proximal tibial adjustment (mm)	2.0	1.5
Valgus	Valgus HKA threshold (°)	3	2
	Lateral distal femur adjustment (mm)	2.0	0.5
	Lateral proximal tibial adjustment (mm)	2.0	1.0

### CPAK parameters

JLO was calculated as MPTA + LDFA, and aHKA was calculated as MPTA—LDFA. JLO and aHKA were calculated from both radiographic observers, from the mean radiographic measurements, and from both wear assumptions for the navigated data. CPAK groups were determined based on the report by MacDessi et al. [11].

Sub-analyses were performed to compare errors in CPAK parameters between LLR and imageless robotic navigation by BMI and preoperative coronal alignment. BMI was split into two groups: ≥ 35 kg/m^2^, and < 35 kg/m^2^, and preoperative alignment was divided into three groups: varus (≥ 3°), valgus (≥ 3°), and neutral (< 3°) groups.

### Statistical analysis

Kolmogorov-Smirnov tests confirmed that MPTA, LDFA, JLO, and aHKA were all normally distributed for both LLR observers, LLR_mean_, Nav_lit_, and Nav_opt_ (*P* > 0.05) [30]. An a priori matched pair two-tailed means analysis was performed using an alpha of 0.05, beta of 0.8, a threshold difference of 1°, and standard deviations of 3.1° and 3.4° from a pilot study, which determined that a minimum of 53 participants were required.

Mean, standard deviation, signed error, RMSE, and mean absolute error (MAE) were calculated for MPTA, LDFA, JLO, and aHKA and compared between LLR observers, between LLR_mean_ measurements and Nav_lit_, and between LLR_mean_ and Nav_opt_.

Two-way mixed effects intraclass correlation coefficients (ICC) were used to report interobserver agreement in CPAK parameters between LLR observers. Welch’s unequal variance *t*-tests were used whenever comparing means, and F-tests were used whenever comparing variances. Bland-Altman plots were used to assess agreement between LLR observers, LLR_mean_ and Nav_lit_, and LLR_mean_ and Nav_opt_, and the percentage of cases within the expected 95% limits of agreement (LOA, i.e., within ± 1.96 SD) were calculated for all CPAK parameters [31, 32]. All analyses were performed using the R environment for statistical computing (version 4.1.0) [33].

## Results

### LLR_1_ vs. LLR_2_

**Fig. 4:**
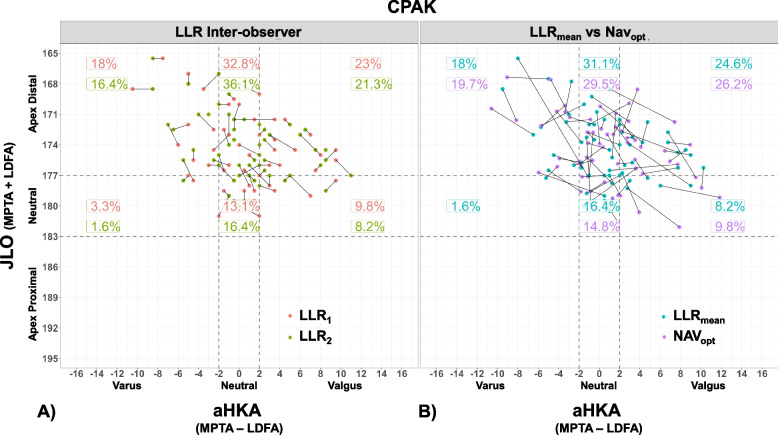
CPAK distribution plots between LLR observers (**a**), and between LLR_mean_ and Nav_opt_ (**b**)

Between LLR observers, all CPAK parameters had highly reliable ICCs (> 0.95), with RMSE below 1.2° (Table 2). No CPAK parameters were significantly different between LLR observers for both means and variance tests. 73% and 97% of patients were within the same and within one CPAK group, respectively, between observers. Figure 4a shows the CPAK distribution for both observers, with joining lines for each patient showing relative change between observers. Because of the excellent agreement between LLR observer measurements, LLR_mean_ values were used to compare against the surgical navigation data.

**Table 2:** CPAK measurements (Mean ± SD) and comparisons between observers, and between LLR_mean_ and both navigation data wear assumptions

		**MPTA**	**LDFA**	**JLO**	**aHKA**
**Measurements**	**LLR**_**1**_	87.8 ± 3.1	86.8 ± 2.3	174.5 ± 3.3	1 ± 4.3
	**LLR**_**2**_	87.4 ± 3.1	86.8 ± 2.1	174.2 ± 3.3	0.6 ± 4.2
	**LLR**_**mean**_	87.6 ± 3.1	86.8 ± 2.2	174.4 ± 3.3	0.8 ± 4.2
	**Nav**_**lit**_	87.6 ± 3.1	87.4 ± 1.9	175.0 ± 3.4	0.3 ± 3.8
	**Nav**_**opt**_	87.7 ± 3.4	86.9 ± 2.2	174.5 ± 3.4	0.8 ± 4.6
**LLR Interobserver** **LLR**_**1**_** vs. LLR**_**2**_	**ICC**	0.963	0.955	0.952	0.965
	**MAE**	0.7	0.5	0.8	1.0
	***t*-test**	0.52	0.86	0.63	0.57
	**F-test**	0.90	0.62	0.88	0.77
**Literature Based** **Nav**_**lit**_** vs. LLR**_**mean**_	**Error**	-0.1 ± 2.6	-0.6 ± 1.7	-0.6 ± 2.9	0.5 ± 3.4
	**ICC**	0.641	0.647	0.636	0.642
	**MAE**	2.0	1.4	2.1	2.7
	***t*-test**	0.94	0.11	0.29	0.48
	**F-test**	0.58	0.77	0.75	0.76
**Optimized** **Nav**_**opt**_** vs. LLR**_**mean**_	**Error**	-0.1 ± 2.5	-0.1 ± 1.6	-0.1 ± 2.9	0 ± 3.1
	**ICC**	0.703	0.734	0.641	0.755
	**MAE**	1.9	1.3	2.2	2.5
	***t*-test**	0.90	0.83	0.83	0.99
	**F-test**	0.53	0.79	0.74	0.53

### Nav_lit_ vs. LLR_mean_

There were no significant differences observed when comparing CPAK parameters between Nav_lit_ and LLR_mean_ for both means and variance tests. Differences in mean values between Nav_lit_ and LLR_mean_ were within 0.6° for all CPAK parameters, with standard deviations below 3.4°. 41% and 88% of patients were within the same and within one CPAK group, respectively when comparing Nav_lit_ and LLR_mean_.

### Nav_opt_ vs. LLR_mean_

Similarly, there were no significant differences found when comparing CPAK parameters between Nav_opt_ and LLR_mean_ for both means and variance tests. Differences in mean values between Nav_opt_ and LLR_mean_ were within 0.1° for all CPAK parameters, with standard deviations below 3.1°. The CPAK distribution for Nav_opt_ and LLR_mean_ is shown in Fig. 4b. 49% and 94% of patients were within the same and within one CPAK group, respectively when comparing Nav_opt_ and LLR_mean_.

**Fig. 5:**
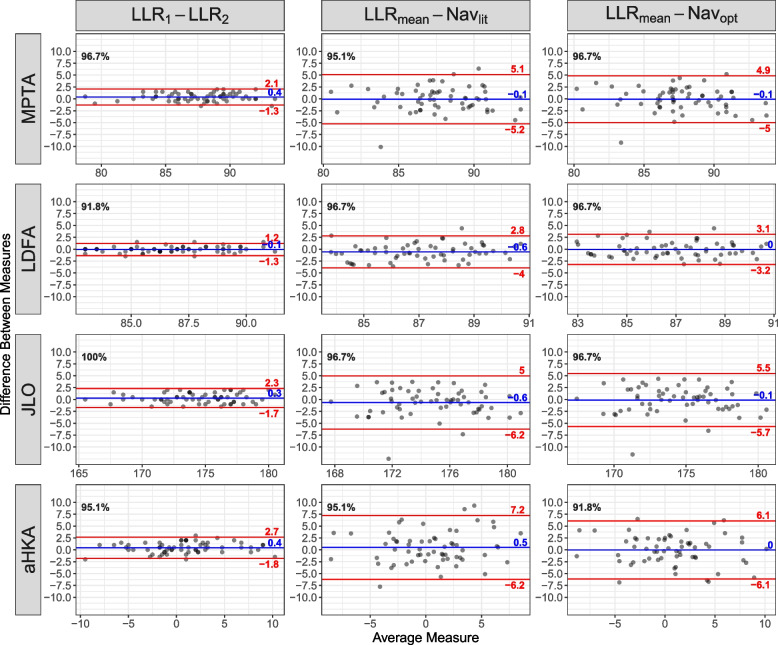
Bland-Altman plots for the various CPAK parameters between LLR observers, and between LLR_mean_ and both Nav_lit_ and Nav_opt_. Percentage (black) values indicate percentage within expected 95% limits of agreement, red values indicate ± 1.96 SD values, and blue values indicate mean difference (bias)

Bland-Altman comparisons yielded > 95% of measurements within the 2 SD limit of agreement for LLR_1_ vs. LLR_2_ and LLR_mean_ vs. Nav_lit_, and > 91.8% for LLR vs. Nav_opt_, indicating good agreement between all measurement methods [31, 32] (Fig. 5).

The BMI sub-analysis indicated no significant differences between the high and low BMI groups for any of the CPAK parameters (*P* > 0.17). Coronal alignment had a significant effect on LDFA and aHKA for Nav_lit_ with valgus knees having higher MAE for LDFA than varus and neutral (2.3° vs. 1.4°, *P* = 0.02 vs. 0.9°, *P* < 0.002). aHKA MAE was also higher for valgus vs. varus knees (4.2° vs. 2.2°, *P* = 0.008) (see supplementary Fig. S1 and Table S1). The difference in MAE for MPTA was not significant for varus, neutral and valgus knees with the numbers available (1.7° vs. 2.3° vs. 2.5°, respectively, *P* > 0.21).

## Discussion

The most important result of this study was that intraoperative imageless robotic navigation data combined with a generic wear assumption of 2 mm was able to determine LLR derived CPAK parameters with a mean difference within 0.6° for all CPAK parameters, and a mean absolute error (MAE) of 2° for MPTA and 1.5° for LDFA. This resulted in MAE for JLO and aHKA of 2.1° and 2.7°, respectively. The accuracy of imageless navigation data for aHKA was comparable, if not better, than previous reports for aHKA using CT-based imaging with robotics and intraoperative stressed measures, which demonstrated standard deviations of 5.3° and 4.2°, respectively [16]. Optimizing the wear parameters led to minor improvements in accuracy.

Personalized alignment strategies that target recreation of native knee joint morphology may limit the need for soft tissue releases and result in more natural kinematics [8, 9, 34, 35]. It is therefore necessary that an operative modality used to execute a personalized knee replacement can accurately determine the constitutional alignment and joint line obliquity of the knee, such that those parameters can then be targeted for the knee reconstruction. There are several available methods for identifying native coronal plane morphology, including generic cartilage wear assumptions, use of long leg radiographs, or methods based on advanced imaging [12–15]. Some of the methods based on CT or MRI use proprietary algorithms that may not be accessible to all surgeons [14–17].

Tarassoli et al. compared radiographic assessments of aHKA using long leg radiographs with CT-based assessments and intraoperative navigation-based assessments of “stressed” HKA based on surgeon application of a deformity-correcting angular force to the knee [16]. They found no significant differences between radiograph-based aHKA, CT-based aHKA, or stressed HKA. Standard deviations for CT-based HKA and stressed HKA, in comparison to radiograph-based aHKA, were 5.3° and 4.2°, respectively. Our standard deviation was 3.4° with literature-based cartilage assumptions, which decreased to 3.1° with optimized cartilage assumptions. Tarassoli et al. reported, in a subsequent study, that long leg radiographs underestimated proximal tibial varus in comparison to CT-based assessments by 1.3° on average [17]. The authors described that the MPTA, as measured from CT images, is sensitive to the sagittal position of the tibial landmarks [17]. Our data are consistent with this observation, which is reflected by increased standard deviations for the MPTA compared to the LDFA for both the literature-based cartilage-assumptions (2.6° vs. 1.7°) and the optimized cartilage-assumptions (2.5° vs. 1.6°). Proximal tibial landmarking that does not fully capture the cartilage loss is particularly relevant to the valgus knee, where wear is often posterolateral, but the landmark is taken in the mid-coronal plane. This likely explains the smaller magnitude of the lateral wear assumptions in the optimized model.

Our study is the first to investigate the accuracy of imageless intraoperative navigation data in determining MPTA and LDFA, which yield the aHKA, joint line obliquity, and overall CPAK class. Our data lend support to the use of this operative modality as a means of identifying the native knee phenotype. The interobserver agreement for MPTA and LDFA measurements from long leg radiographs was very strong, which supports use of long leg radiographs as the gold standard reference for this analysis. Use of aHKA to predict constitutional alignment does assume that the distal femur and proximal tibia joint surfaces in the native knee are parallel. In fact, prior study has shown this to be the case within mean 0.5° and standard deviation 1° [10]. Further study to directly compare CT-based and imageless navigation-based assessments of CPAK parameters is warranted.

We utilized two sets of assumptions regarding cartilage loss. The first set of assumptions relied on prior literature reports of the thickness of lost cartilage of knees with preoperative varus or valgus deformities [6, 24, 29]. In large cohorts of non-arthritic knees undergoing MRI, cartilage thickness has been reported to be in the 1.5–2.0 mm range [36, 37]. In a study of over 200 arthritic knees that underwent MRI imaging, Nam et al. reported a mean distal femoral cartilage wear of 1.7 mm medially for varus knees and 1.3 mm laterally for valgus knees [29]. Importantly, they also reported that over 99% of arthritic knees had < 1 mm of bone wear [29]. Our second set of assumptions was optimized to this patient cohort via parametric analysis. Our optimized wear assumption predicted less wear on the lateral side on both the tibia and femur for valgus deformities. This finding agrees with data from Nam et al., which suggests that there may be less than 2 mm of wear on most valgus knees [29], and also may reflect the landmarking algorithm used with this imageless navigation system.

This study had several limitations. First, accuracy of imageless navigation is dependent upon the quality of the registration, and results presented here could be different if appropriate registration is not performed. Second, the cartilage assumptions used in NAV_opt_ were optimized to this cohort of patients and may not be generalized to other patient populations. While the patient population in this study had a large range of coronal deformity from 16° valgus to 17° varus, the accuracy in determining CPAK parameters for both radiographic and imageless robotics methods may differ for more severe deformities outside of this range where bone wear may be more pronounced. In a matched-pairs study comparing aHKA in the osteoarthritic knee to mechanical HKA in the contralateral healthy knee, Macdessi et al. found a 1.3° greater mean difference between measurements for knees with coronal deformities > 8° using the LLR method [38]. A similar phenomenon may be true for robotic data as bone erosion can alter the landmarks used to determine LDFA and MPTA and we therefore recommend exercising caution when selecting landmarks to calculate CPAK parameters for patients with significant deformity and bone wear. Additionally, we found that valgus knees tended to have higher MAE than varus knees, which may indicate that the degree of wear is less predictable in valgus than varus deformities [39]. Lastly, our study was powered to determine a mean difference of 1° between methods and, as such, had a relatively small sample size of 61 patients. Therefore, not all CPAK phenotypes may be sufficiently represented. However, a 1° difference was determined clinically sufficient to power our study and similar patient numbers have been used in other studies comparing LLR to CT for CPAK [16, 18]. Fourth, our study lacked clinical outcomes data, and application of CPAK parameters to alignment strategies in TKA requires additional study.

## Conclusion

The results from this investigation indicate that imageless intraoperative robotic navigation data can be used to determine CPAK parameters for arthritic knees undergoing TKA when correcting for wear, with similar CPAK values obtained to those determined from LLR. Computer-assisted imageless TKA systems should consider incorporating CPAK planning into their workflows using the intraoperative landmark data.

### Supplementary Information

**Additional file 1: Fig. S1. **Distribution and significance of absolute (a) and signed (b) error in CPAK parameters by preoperative alignment group for Nav_lit_ and Nav_opt_ vs LLR_mean_. **Table S1.** a) Mean Absolute Error (MAE), and b) signed error for MPTA, LDFA, JLO, and aHKA split by preoperative coronal deformity for Nav_lit_ and Nav_opt_.

## Data Availability

The datasets used and/or analysed during the current study are available from the corresponding author on reasonable request.
