# Supplementary material for: Imageless robotic total knee arthroplasty determines similar coronal plane alignment of the knee (CPAK) parameters to long leg radiographs
Source: Arthroplasty. 2024 Mar 3;6:14. doi: 10.1186/s42836-024-00231-9 (PMC10909262; doi:10.1186/s42836-024-00231-9)
Supplement: Supplementary file 1 — Additional file 1: Fig. S1. Distribution and significance of absolute (a) and signed (b) error in CPAK parameters by preoperative alignment group for Navlit and Navopt vs LLRmean. Table S1. a) Mean Absolute Error (MAE), and b) signed error for MPTA, LDFA, JLO, and aHKA split by preoperative coronal deformity for Navlit and Navopt. [file 42836_2024_231_MOESM1_ESM.docx]

**Fig. S1.** Distribution and significance of absolute (a) and signed (b) error in CPAK parameters by preoperative alignment group for Nav_lit_ and Nav_opt_ vs LLR_mean_. Statistically significant differences are indicated in figures by “*” = P < 0.05; “**” = P < 0.01; “***” = P < 0.001. Boxplots represent medians, with lower and upper boxes corresponding to the first and third quartiles (25th and 75th percentiles), lower and upper whiskers extending to the smallest and largest values within 1.5 * IQR (inter-quartile range, or distance between the first and third quartiles), and data beyond the whiskers plotted individually as outlying points.

a)
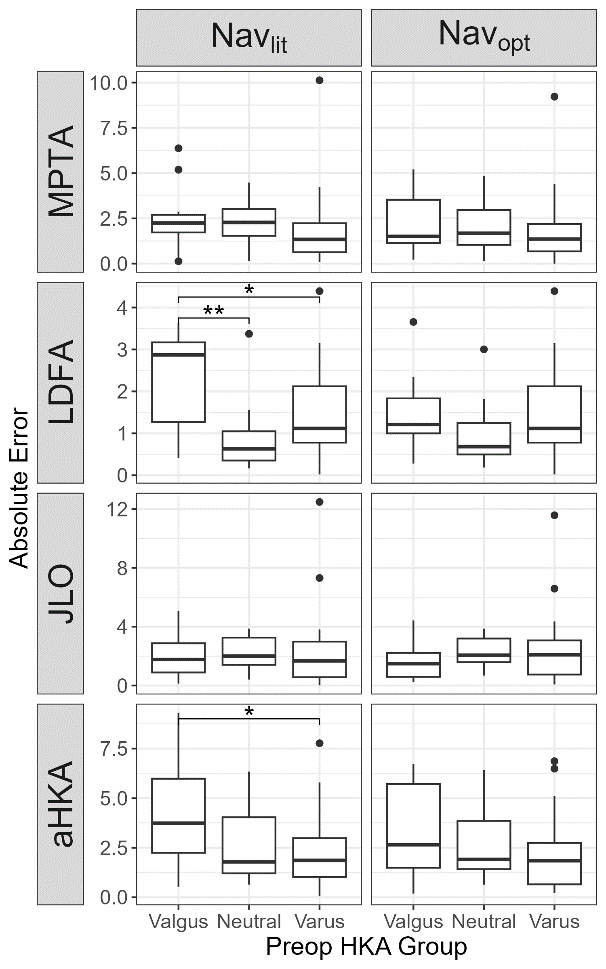
 b)
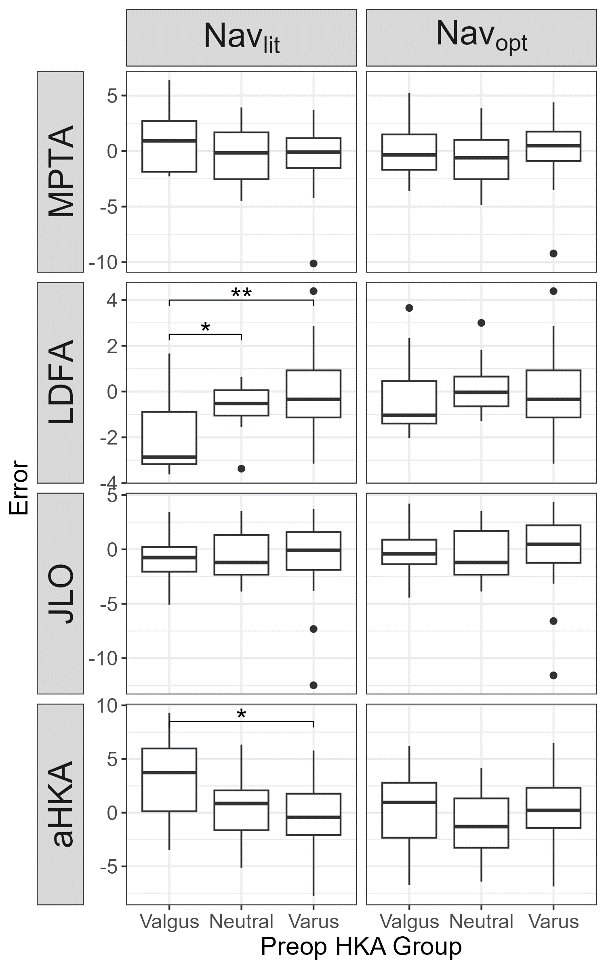


**Table S1.** a) Mean Absolute Error (MAE), and b) signed error for MPTA, LDFA, JLO, and aHKA split by preoperative coronal deformity for Nav_lit_ and Nav_opt_

| a) **MAE** | **Nav_lit_** | | | **Nav_opt_** | | |
| --- | --- | --- | --- | --- | --- | --- |
|  | **Valgus (≤-3°)** | **Neutral** | **Varus (≥3°)** | **Valgus (≤-3°)** | **Neutral** | **Varus (≥3°)** |
| MPTA | 2.5 | 2.3 | 1.7 | 2.3 | 2.1 | 1.7 |
| LDFA | 2.3 | 0.9 | 1.4 | 1.5 | 1.0 | 1.4 |
| JLO | 1.9 | 2.2 | 2.2 | 1.8 | 2.3 | 2.2 |
| aHKA | 4.2 | 2.7 | 2.2 | 3.3 | 2.6 | 2.2 |

| **b) Signed error** | **Nav_lit_** | | | **Nav_opt_** | | |
| --- | --- | --- | --- | --- | --- | --- |
|  | **Valgus (≤-3°)** | **Neutral** | **Varus (≥3°)** | **Valgus (≤-3°)** | **Neutral** | **Varus (≥3°)** |
| MPTA | 1.1 | -0.1 | -0.4 | 0.1 | -0.8 | 0.2 |
| LDFA | -1.9 | -0.6 | -0.1 | -0.3 | 0.3 | -0.1 |
| JLO | -0.8 | -0.7 | -0.5 | -0.2 | -0.5 | 0.1 |
| aHKA | 3.0 | 0.5 | -0.4 | 0.3 | -1.0 | 0.3 |
